# *In vitro* induction and characterisation of tetraploid drumstick tree (*Moringa oleifera* Lam.)

**DOI:** 10.1515/biol-2020-0087

**Published:** 2020-11-18

**Authors:** Junjie Zhang, Ruiqi Pian, Endian Yang, Wei Zhou, Qian He, Xiaoyang Chen

**Affiliations:** State Key Laboratory for Conservation and Utilization of Subtropical Agro-bioresources (South China Agricultural University), Guangzhou, 510642, China; Guangdong Key Laboratory for Innovative Development and Utilization of Forest Plant Germplasm, Guangzhou, 510642, China; Guangdong Province Research Center of Woody Forage Engineering Technology, Guangzhou, 510642, China; College of Forestry and Landscape Architecture, South China Agricultural University, Guangzhou, 510642, China

**Keywords:** drumstick tree, colchicine, tetraploid, morphological characteristics, nutritional contents

## Abstract

Artificial induction of polyploidy is widely used in breeding programmes to improve the agronomic traits. The drumstick tree (*Moringa oleifera* Lam.) has a range of potential commercial uses, as the vegetative organs have high nutritional, medicinal, and feed values. In the present study, *in vitro* tetraploidisation in drumstick tree was performed by treating leaf segments with colchicine and subsequently verifying the ploidy levels. For polyploidisation, explant survival and regeneration rates were affected more by exposure time than by colchicine concentration, and the highest polyploidisation efficiency was observed at 500 mg/L colchicine for 3 days, which yielded 21% tetraploids. The morphological characteristics and contents of seven fodder-related nutrients (crude protein, ether extract, ash, acid detergent fibre, neutral detergent fibre, calcium, and phosphorus) in the leaves and shoots were compared between tetraploid and diploid drumstick trees. The resulting tetraploids showed significantly enhanced leaf and stomatal size. In addition, the contents of seven fodder-related nutrients were higher, although to varying degrees, in tetraploids than in diploids. The results indicated that the tetraploid produced in this study exhibited superior agronomical traits and improved biomass yield than diploids, and may represent excellent raw materials for fodder to enhance biomass and nutrition.

## 1 Introduction

Polyploidisation is a natural phenomenon in many plants and a major force in plant genome evolution [1]. Comparative genomic analysis and large expressed sequence tag datasets have shown that all angiosperms share at least two polyploidy events in their common evolutionary history [2,3]. Polyploids, commonly defined as having more than two sets of chromosomes in a single cell, are usually superior to diploids in terms of morphological changes and genetic adaptability and have been identified as an important tool for the generation of innovative germplasm resources and plant breeding [1,4,5]. Polyploids can be valuable resources for medicinal and feed plants with functional compounds in vegetative tissue, because they often exhibit increased biomass or concentrations of effective compounds [6,7,8]. Therefore, induction of polyploidy is a useful strategy for crop improvement. Among many available induction methods, colchicine application has been adopted successfully to double the chromosome number in many species [8,9,10].

Drumstick tree (*Moringa oleifera* Lam.) is one of the most well-known and widely planted species in the monogeneric family Moringaceae [11,12]. This species is indigenous to the sub-Himalayan areas of India and various tropical African countries [11]. Because of its high commercial value, interest in its cultivation has been extended to many tropical and subtropical countries [11,13]. Drumstick tree is considered to be one of the most useful trees in the world because almost all parts of this plant have various medicinal properties linked to antimicrobial [14], anti-inflammatory [15], detoxification [16] and anticancer activities [17], and high nutritional values, enabling their use as vegetables, in medicine, and for industrial purposes [18,19].

Drumstick tree is a diploid with a chromosome number of 2*n* = 2*x* = 28 in somatic cells [20]. To support product development and improve raw material quality by creating superior drumstick tree cultivars, it is necessary to develop drumstick tree tetraploid plants using tissue culture techniques. Therefore, in this study, we used colchicine treatment to obtain drumstick tree polyploids from leaf explants. The objectives of this study were to investigate the effects of colchicine on the induction of tetraploidy in drumstick tree and to compare the characteristics of tetraploid trees with diploid trees.

## 2 Materials and methods

### 2.1 Culture media and growth conditions

All media used were adjusted to pH 5.8–6.0 with 1 N NaOH or 1 N HCl solution, set using 0.45% (w/v) agar except for liquid medium, and autoclaved at 121°C for 15 min. Cylindrical, transparent polypropylene containers (10 cm height × 7 cm diameter) with an internal volume of 280 mL were used for all cultures. Each bottle was filled with 40 mL of medium and covered with a polycarbonate screw cap so that they were nearly air-tight. All cultures were kept under cool white light (about 50 µmol m^−2^ s^−1^) with a 12-h photoperiod and a temperature of 25–27°C unless otherwise stated.

### 2.2 Plant material and tetraploid induction

Donor plants in this study were *in vitro*-grown shoots of clonal drumstick tree M-2. Stage 2 leaves from M-2 were wounded and pre-cultured on shoot regeneration medium, which consisted of Murashige and Skoog basal medium supplemented with 0.8 mg/L 6-benzyladenine, 0.2 mg/L kinetin, and 0.05 mg/L 1-naphthaleneacetic acid, for 3 days to heal the cutting wound and initiate cell division [13]. Then, the explants were transferred to the same liquid medium supplemented with 100 mg/L colchicine for different durations (3, 6, 9, and 12 days) and cultured with constant shaking at 50 rpm under dark conditions. Alternatively, the explants were exposed to liquid medium supplemented with different concentrations (0, 100, 250, 500, and 1,000 mg/L) of colchicine for 3 days. After colchicine treatment, the treated explants were transferred to solid shoot regeneration medium without colchicine to induce shoot development. Each treatment consisted of 15 replicates and was repeated three times. Regenerated shoots reaching 2 cm in height were cut from the mother tissues and cultured in the rooting medium to initiate the root growth and further growth of the intact regenerated plantlets.

### 2.3 Chromosome observation and counting

The chromosome number was identified using the squashing method of Nilanthi with slight modifications [21]. Actively growing root tips 5–10 mm in length from each regenerated plant were excised. These root tips were treated with ice water for 4 h and fixed in Carnoy’s solution for 24 h at room temperature. The fixed root tips were hydrolysed in 1 N HCl for 10 min at 65°C, after which root tips were washed with tap water for 20 min and cut into shorter root tip segments ∼1.5 mm in length. The prepared root tips were placed on glass slides, stained with one drop of carbol fuchsin solution for 30 min, squashed under a cover glass, and observed for their chromosome numbers under a microscope (Olympus BX43) through a 100× objective lens. Photographs were taken with the associated camera. Plants with all root tip cells containing 28 chromosomes were determined to be diploid, those with some cells containing 28 and other cells containing 56 chromosomes were determined to be chimeras, and those with all cells containing 56 chromosomes were determined to be tetraploid.

### 2.4 Evaluation of stomatal and leaf characteristics of tetraploid plants

The stomatal and leaf characteristics of tetraploid and diploid counterparts were evaluated. Relatively mature leaves of diploid and tetraploid *ex vitro* plants were used to compare differences in stomata. Several excised pieces of epidermal layer from the abaxial side of leaves were mounted on glass slides with a drop of distilled water and covered with a cover slip [6,22]. The density of stomata was measured under a light microscope under a 20× objective lens, with 30 randomly selected microscopic fields per sample. The size (length) of the stomata was measured under a 100× objective lens, and 100 randomly selected stomata were used to calculate the stomatal size. The leaf characteristics, including leaf length, width, and leaf shape index, during the growing period of 1-month-old plants were compared between tetraploid and diploid plants. The values from ten plants were used to evaluate the selected characteristics.

### 2.5 Characterisation of fodder-related nutritional composition of tetraploid plants

The fodder-related nutritional compositions of tetraploid and diploid counterparts were evaluated. We analysed crude protein (CP), ether extract (EE), ash, neutral detergent fibre (NDF), calcium (Ca), and phosphorus (P) following the methods of the Association of Official Analytical Chemists [23] and acid detergent fibre (ADF) using the method described by Van Soest et al. [24]. Three duplicate assays were performed for each characteristic.

### 2.6 Statistical analysis

All experiments were arranged in a randomised complete block design. Statistical analysis was carried out using SPSS ver. 19.0 software (SPSS Inc., Chicago, IL, USA). Duncan’s multiple range test was used to detect differences among the mean values. Results with *P* values < 0.05 were considered to be significant.

## 3 Results and discussion

### 3.1 Tetraploid induction and verification

**Figure 1:**
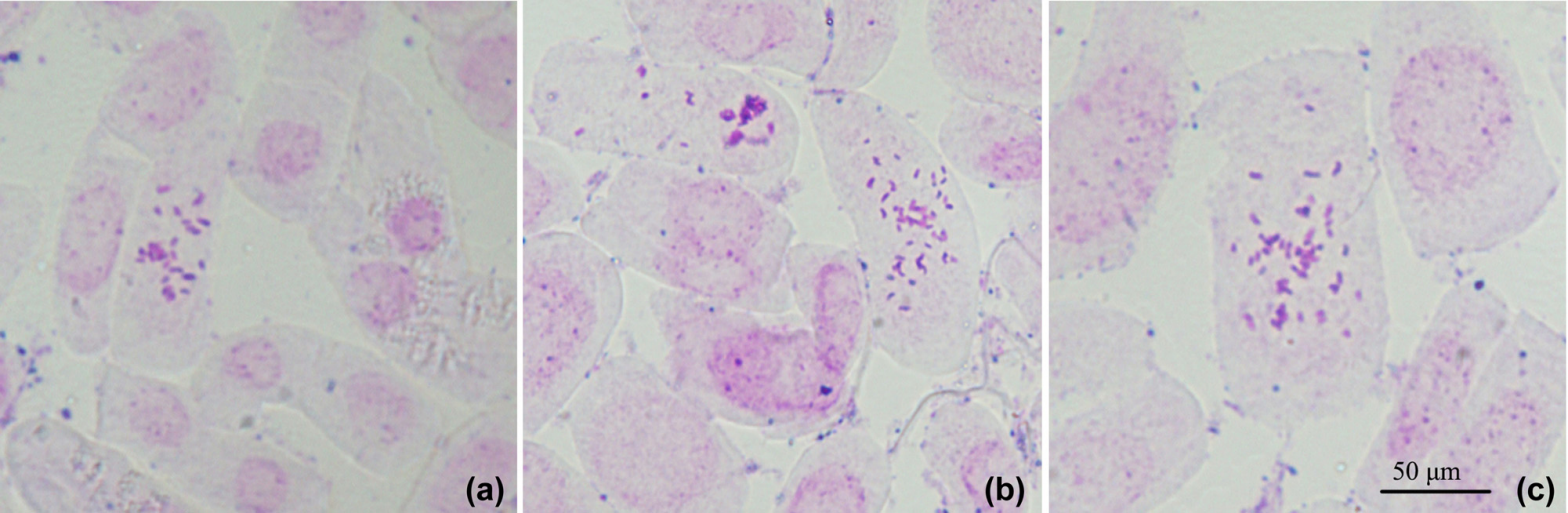
Chromosomes in the root tips of drumstick tree: (a) diploid, (b) chimeras, and (c) tetraploid.

Colchicine is a highly effective mitotic spindle inhibitor that has been used with great success to improve the agronomic traits of a number of species via polyploid induction [4,6,22]. In this study, we investigated the effects of exposure time and colchicine concentration on tetraploid induction. The leaves of drumstick tree proved to be very sensitive to colchicine exposure, and prominent cytotoxic effects were observed after the treatment. The survival and regeneration rates decreased substantially with increasing concentration and exposure time (Tables 1 and 2), similar to the results of Hannweg et al. [25] and Zhang et al. [10]. Moreover, the survival and regeneration rates were affected more by exposure time than by colchicine concentration (Tables 1 and 2). Following colchicine treatment, it is important to identify the ploidy levels of regenerated plant lines. In this study, the ploidies were determined by chromosome counting (Figure 1). Tables 3 and 4 summarise the chromosome-counting results. The results showed that both tetraploids and chimeras were induced in the treatment. The most effective treatment for inducing polyploidy was 500 mg/L colchicine treatment for 3 days, which yielded 21% tetraploids. Exposure time had the greatest effect on the tetraploid induction rate, possibly due to the lower survival rate of explants after overexposure to colchicine. Chimera induction always accompanies tetraploid induction and has been reported in many species, such as *Echinacea purpurea* [21], *Liquidambar styraciflua* [10], *Dendrobium officinale* [26], and so on.

**Table 1:** Effect of different exposure time of 100 mg/L colchicine treatment on shoot regeneration in drumstick tree

Exposure time (days)	% explants regenerated shoots	Shoots per explant
0	82.43 ± 10.28a	3.43 ± 0.62a
3	32.0 ± 6.66b	0.60 ± 0.14b
6	16.74 ± 4.73bc	0.36 ± 0.21b
9	9.46 ± 4.02c	0.12 ± 0.04b
12	5.42 ± 3.33c	0.04 ± 0.01b

Mean values followed by the same letter in the same column are not significantly different from each other at the *P* ≤ 0.05 level, according to Duncan’s multiple range test.

**Table 2:** Effect of different colchicine concentration *in vitro* treatments on shoot regeneration in drumstick tree

Colchicine (mg/L)	% explants regenerated shoots	Shoots per explant
0	82.43 ± 10.28a	3.43 ± 0.62a
100	32.0 ± 6.66b	0.60 ± 0.14b
250	21.67 ± 5.36bc	0.49 ± 0.10bc
500	17.50 ± 6.08bc	0.43 ± 0.19bc
1,000	6.67 ± 4.63c	0.07 ± 0.05c

Mean values followed by the same letter in the same column are not significantly different from each other at the *P* ≤ 0.05 level, according to Duncan’s multiple range test.

**Table 3:** Effect of different exposure time of 100 mg/L colchicine treatment on polyploid induction in drumstick tree

Exposure time (days)	No. of plants examined	% individuals by ploidy level		
		Diploid	Chimeras	Tetraploid
0	10	100a	0c	0c
3	27	81.48 ± 7.62b	14.81 ± 6.97ab	3.70 ± 3.70b
6	16	68.75 ± 11.97c	25.0 ± 11.18ab	6.25 ± 3.39b
9	6	50.0 ± 22.36c	33.33 ± 21.08a	16.67 ± 5.94a
12	2	100a	0c	0c

Mean values followed by the same letter in the same column are not significantly different from each other at the *P* ≤ 0.05 level, according to Duncan’s multiple range test.

**Table 4:** Effect of different colchicine concentration *in vitro* treatments on polyploidy induction in drumstick tree

Colchicine (mg/L)	No. of plants examined	% individuals by ploidy level		
		Diploid	Chimeras	Tetraploid
0	10	100a	0c	0c
100	27	81.48 ± 7.62b	14.81 ± 6.97b	3.70 ± 3.70c
250	22	63.64 ± 10.50b	27.27 ± 9.72a	9.09 ± 6.27b
500	19	52.63 ± 11.77c	26.32 ± 10.38a	21.05 ± 9.61a
1,000	3	66.67 ± 33.33b	33.33 ± 33.33a	0c

Mean values followed by the same letter in the same column are not significantly different from each other at the *P* ≤ 0.05 level, according to Duncan’s multiple range test.

### 3.2 Morphological characterisation of tetraploid drumstick tree plants

**Figure 2:**
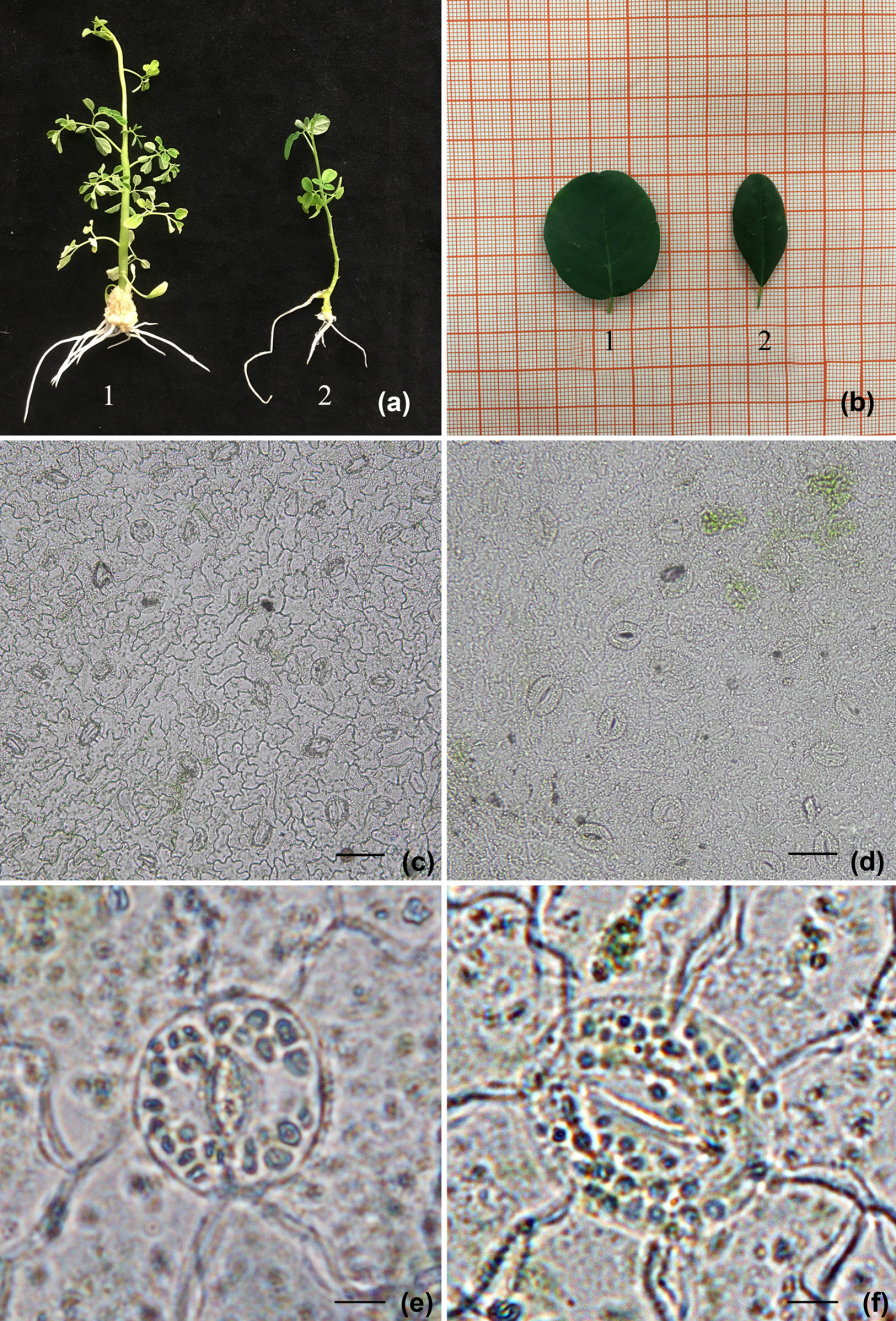
Comparison of morphology and stomata between diploid and tetraploid drumstick tree plants. (a) Plantlets of tetraploid (1) and diploid (2) drumstick tree. (b) Leaves of tetraploid (1) and diploid (2) drumstick tree. (c and e) Stomata of diploid plants. (d and f) Stomata of tetraploid plants. Bar = 50 µm (c and d) and 10 µm (e and f).

A number of studies have demonstrated that polyploid plants can vary in their morphology, ecology, physiology, and cytology compared to the parental line [1,6,9,27]. Such variations have been used successfully in plant breeding programmes to develop superior cultivars [8,28,29,30]. Here, we assessed a range of selected characteristics to identify significant differences between the diploid and tetraploid plants. The morphological characteristics of the tetraploid drumstick tree leaves differed significantly from those of their diploid counterparts. The tetraploids had obviously larger size (Figure 2a) and leaves (Table 5 and Figure 2b) than the diploids. In addition, the leaf shape index (leaf length/width) was lower in the tetraploid plants than the diploid plants (Table 5). The stomatal density of the leaves on the tetraploid plants was lower and the size was larger than that of diploid plants (Table 5 and Figure 2c–f). In addition, the tetraploids had nearly twice the number of chloroplasts in the guard cells (Figure 2e and f). The larger mean size of the tetraploid stomata than diploid stomata suggested that the tetraploids had larger cells.

**Table 5:** Morphological and stomatal comparisons of diploid and tetraploid drumstick tree

Characteristics	Diploid	Tetraploid
Leaf length (cm)	2.08 ± 0.05	2.95 ± 0.19
Leaf width (cm)	1.15 ± 0.04	2.37 ± 0.16
Leaf shape index (leaf length/width)	1.82 ± 0.03	1.26 ± 0.08
Stomatal length (µm)	27.10 ± 0.72	41.90 ± 0.64
Stomatal density (N/mm^2^)	151.73 ± 3.42	93.47 ± 1.49

Analysing the morphology of diploid and tetraploid drumstick tree plants revealed typical features of polyploids. Polyploidy affects plant developmental rate and, frequently, the overall size of cells, organs, and the entire organism [1,27,31,32]. Chromosome doubling translates into doubling the number of genes, and this can increase the gene expression level associated with a particular beneficial feature [33,34]. The increases in stomatal and leaf size might have been due to increases in cell size, while genetic changes in cells might have enhanced the gene activity.

### 3.3 Comparison of fodder-related nutritional composition between diploid and tetraploid drumstick tree plants

The CP, EE, ash, NDF, ADF, Ca, and P contents of plants are important indicators for evaluating the nutritional values of forage. Therefore, we examined the contents of these seven fodder-related nutritional components. Table 6 presents the results. The contents of all seven nutritional components were higher, albeit to different degrees, in tetraploid plants. The analysis indicated that chromosome duplication had a significant influence on bioactive compound concentrations. This phenomenon in tetraploids has been reported for other plant species. For example, tetraploid *Anoectochilus formosanus* Hayata had significantly higher gastrodin and flavonoid contents than diploids [6]. And, most of the tetraploid *Sophora tonkinensis* Gapnep. lines exhibited higher productivity of total contents of matrine and oxymatrine than diploids [35]. In addition, tetraploid *E. purpurea* had higher caffeic acid derivative and alkamide contents [8]. Finally, tetraploid *Thymus persicus* Jalas plants showed a significant increase in triterpenoids compared to diploid plants [31]. Polyploidy does not simply result in chromosome doubling and is also accompanied by changes in genome structure and gene expression [36,37]. In addition, genomic plasticity has downstream effects on the transcriptome, proteome, and metabolome that can generate phenotypic variations in polyploids that exceed the performance of the parents [38,39]. However, detailed mechanisms associated with variations in the chemical composition due to polyploidisation remain unclear. Understanding these mechanisms will help promote breeding programmes.

**Table 6:** Fodder-related quality comparisons (g/kg) in leaves of diploid and tetraploid drumstick tree

Nutrition composition	Diploid	Tetraploid
Crude protein	262.30 ± 29.91	325.94 ± 40.20
Crude fat	40.43 ± 4.73	45.69 ± 3.34
Crude ash content	90.61 ± 4.80	119.61 ± 6.57
Acid detergent fibre	128.98 ± 2.61	149.55 ± 1.08
Neutral detergent fibre	153.08 ± 12.78	187.82 ± 14.70
Calcium	16.42 ± 1.71	23.15 ± 1.94
Phosphorus	3.45 ± 0.52	4.68 ± 0.62

## 4 Conclusion

In this study, we obtained tetraploid drumstick tree plants from colchicine-treated leaf explants. This protocol is effective, not limited by flowering period, and relatively simple. The duplication of genetic information was correlated with variations in the traits investigated in this species. The tetraploid plants had larger leaves and stomata and exhibited higher nutritional values than the original diploid plants. Additional research is required to determine the detailed effects of polyploidy on harvest yield and nutritional composition under field conditions. The tetraploid drumstick tree plants induced in this study are promising materials for obtaining a superior variety with high biomass and nutritional values.
